# Graphs in the COVID-19 news: a mathematics audit of newspapers in Korea

**DOI:** 10.1007/s10649-021-10029-0

**Published:** 2021-03-04

**Authors:** Oh Nam Kwon, Chaereen Han, Changsuk Lee, Kyungwon Lee, Kyeongjun Kim, Gyeongha Jo, Gangwon Yoon

**Affiliations:** 1 Department of Mathematics Education, Seoul National University, Gwanak-ro 1, Gwanak-gu, Seoul, 08826 South Korea; 2 Graduate School of Seoul National University, Gwanak-ro 1, Gwanak-gu, Seoul, 08826 South Korea

**Keywords:** Graph, Graph usage, Misuse of graph, COVID-19 news, South Korea

## Abstract

Visual displays in the news media become critical during escalating events such as the COVID-19 pandemic, as they facilitate the communication of complex information to the public. This article investigates the use of graphs in Korea’s news media during the COVID-19 outbreak. We selected 12 dates that represent turning points in the outbreak of the disease and collected news stories including graphs from seven Korean daily newspapers issued on those dates. First, we analyzed the usage of graphs in COVID-19 news stories. Quantitative analysis of the types and frequency of graphs used in COVID-19 news stories and qualitative analysis of the content of news stories containing graphs were conducted. Second, we identified cases in which readers may be biased by the mathematical misuse of graphs in the news stories covering COVID-19. The implications of these findings for future teaching and learning of graph literacy in school mathematics courses are discussed.

## Introduction

In current society, print media commonly use graphs to illustrate journalistic arguments in publications, including newspapers, that provide news and information for the general public (Monteiro & Ainley, 2007). This means that the mode of using graphs in newspapers could have an influence on the public, especially when there is a state of international emergency, such as that due to COVID-19. Although research on graphs in newspapers has been conducted mostly in the journalism field (e.g., Dick, 2015), mathematics education researchers also need to pay attention to the way graphs are used in newspapers. This could indicate the future direction of statistical graph education, which should cultivate citizens who can make reasonable decisions. Therefore, in this article, we investigate graph usage in Korean newspapers during the COVID-19 outbreak.

### News of the COVID-19 era

Since the first COVID-19 case in Korea was confirmed on January 20, 2020, newly confirmed cases have soared, and virus transmission has affected the nation through the local and community levels (Park et al., 2020). Information related to COVID-19 affects public behavior, such as preventing further transmission through social distancing and making improvements in personal hygiene (Kim, Seo, & Jung, 2020). As noted in countries with a decline in the pandemic, public involvement in preventing the spread of infectious disease is a major, and potentially the only, solution to curb the spread of COVID-19. The visual display of information provided by the various news media, the major sources through which the public accesses pandemic-related information, has played an increasingly important role in facilitating the communication of complex information on topics such as politics, society, and the economy in modern society (Ancker, Senathirajah, Kukafka, & Starren, 2006; Gracia-Retamero & Cokely, 2013, 2017; Spiegelhalter, Pearson, & Short, 2011). One of the typical visual displays of information is a graph that illustrates numerical data (Shaughnessy, 2007). In this study, we focus on how graphs are used or misused in newspapers in the era of COVID-19. News, including graphs, has an impact on the public understanding of the pandemic, which can ultimately affect the level of disease prevention across the community by guiding individuals’ behaviors regarding preventive measures. Therefore, by examining the use and misuse of graphs in news stories related to COVID-19 in Korea, we aim to draw out the implications of the ability to understand graphical information, called graph literacy, which is required by graph users for rational decision-making.

### Research on the understanding of graphs in mathematics education

Basic graph sense, such as reading within and beyond graphs, is critical for statistical thinking, reasoning, and literacy and, in this regard, has received continued attention from mathematics education researchers (Shaughnessy, 2007). The work of Friel, Curcio, and Bright (2001) is noteworthy, as they carried out a foundational study with a special focus on statistical graphs, not on algebra or calculus. They considered questioning (i.e., asking and posing questions) as a significant aspect of graph comprehension. Additionally, they defined the term graph comprehension as the ability of graph readers to derive meaning from graphs created by others or themselves. Several authors (Bertin, 1983; Carswell, 1992; Curcio, 1981a, 1981b, 1987; McKnight, 1990; Wainer, 1992) have characterized the kinds of questions that graphs are needed to answer. Friel et al. (2001) reported three levels of graph comprehension: an elementary level that is focused on extracting data from a graph (i.e., locating or translating); an intermediate level, characterized by interpolating and finding relationships in the data shown on a graph (i.e., integrating or interpreting); and an advanced level that requires extrapolating from the data and analyzing the implicit relationships in a graph (i.e., generating or predicting). At the advanced level, questions provoke the comprehension of students on the core structure of the data presented. The literature on the levels of graph comprehension highlights that members of the general public might have poor graphical interpretation skills and are often unable to reason beyond the information in graphs. These studies also provide a framework for examining the qualitative aspect of graph usage in COVID-19 news stories from mathematical perspectives. Specifically, two research questions are posed:

- First, how are graphs related to COVID-19 used in news coverage in Korea?
- Second, how are these graphs misused in the news coverage related to COVID-19 in Korea?

## Methods

### South Korea context

In Korea, a country geographically adjacent to China, a 35-year-old Chinese person who arrived at Incheon Airport from Wuhan, China, on January 20, 2020, was diagnosed with the first confirmed case of COVID-19. At that time, the Korea Centers for Disease Control raised the national infectious disease alert level from Level 1 Blue (Attention) to Level 2 Yellow (Caution). On January 27th, with the 4th confirmed case of COVID-19, the government raised the infectious disease alert level to Level 3 Orange (Alert). Subsequently, the number of confirmed cases at Daenam Hospital in Cheongdo, North Gyeongsang Province, underwent an exponential increase in daily confirmed cases. As a measure to address this situation, on February 23rd, the government raised the alert level to the highest level, Level 4 Red (Critical) and substantially strengthened the response system. Korea was ranked as the country with the second-highest number of confirmed cases of COVID-19 in the world, after China, for a total of 19 days, from February 20th to March 9th (World Health Organization, 2020). The number of confirmed cases, which showed a rapid increase after February 18th, exhibited a decreasing trend beginning on March 11th. However, the mass infections in the Guro-gu Call Center and Seongnam Grace River Church raised concerns over the spread of the epidemic in metropolitan areas. From the middle of March, as the number of confirmed cases in Europe and the USA surged, the number of confirmed cases from the overseas influx, comprising students studying abroad in those regions and residents from the regions, increased. Therefore, beginning on March 22nd, Incheon International Airport began testing all incoming travelers from Europe and Chinese travelers for COVID-19 (The Government of the Republic of Korea, 2020). On March 24th, a testing center was installed at Incheon International Airport to screen susceptible individuals with symptoms of COVID-19 among all incoming travelers. As of May 20th, Korea was ranked 45th in terms of the number of confirmed cases of COVID-19 (Hopkins, n.d.) and was considered to have had some degree of success in the prevention of this infectious disease without a national shutdown or lockdown (Chen & Qiu, 2020). Although the number of new confirmed cases per day decreased to approximately 10, the country maintained the alert level at Level 4 Red (Critical) and ordered the continuation of social distancing in everyday life.

### Data collection

**Fig. 1:**
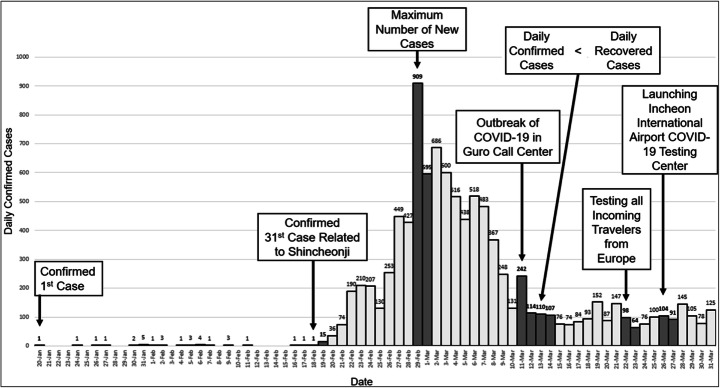
Daily confirmed cases of COVID-19 and critical events

To look into the graphical representation in the news during the outbreak of COVID-19 in Korea, we selected seven daily newspapers from among the top 10 Korean newspapers by circulation based upon accessibility to news articles (*The Chosun Ilbo*, *Korea JoongAng Daily*, *The Dong-A Ilbo*, *The Kukkmin Ilbo*, *Seoul Newspaper*, *The Hankyoreh*, and *Hankook Ilbo*) (Korea Audit Bureau of Circulations, 2020). The stories covering COVID-19 using graphs were closely related to the outbreak trend of the disease. Therefore, critical events that served as turning points in the outbreak of the disease were selected as focal dates rather than targeting all dates after December 31, 2019, when COVID-19-related news was first reported in Korea. News stories published on each focal date and the following date were analyzed. The criterion for selecting the turning points was based on the trend in the number of newly confirmed cases per day as well as dates when the number of recovered or susceptible cases drastically increased or decreased or a new source of community infection was identified. From January 20, 2020, to the end of April 2020, when this study began, six critical events were determined. These dates are as follows (see the arrows in Fig. 1).

- February 18, 2020 (initiation of local infection): With the emergence of the 31st confirmed case related to the Sincheonji church community, infection escalated to local infection levels in the Daegu and Gyeongbuk provinces.
- February 29, 2020 (peak point): The maximum number of daily confirmed cases of COVID-19 occurred.
- March 11, 2020 (reignition of local infection): There was increasing concern about the spread of local infection in the Guro-gu Call Center in Seoul, the capital of Korea.
- March 13, 2020 (golden cross): For the first time, the number of daily recovered cases exceeded the number of daily confirmed cases.
- March 22, 2020 (testing all incoming travelers from Europe): COVID-19 testing began for all incoming travelers from Europe, including Italy and Spain, regions with an increase in the number of confirmed cases of COVID-19, to prevent the spread of COVID-19 due to overseas inflow.
- March 24, 2020 (establishment of the COVID-19 testing center in Incheon International Airport): As the number of confirmed cases in the USA increased drastically, public opinion emerged that entrants from the USA should also be tested. Accordingly, a testing center was installed in Incheon International Airport for disease prevention by screening susceptible individuals with symptoms among all incoming travelers.

In addition to these six critical event dates, the day after each critical event day was also searched for stories covering the effects of these events. Accordingly, the subject of our analysis grew to a set of 5924 news stories published in seven major daily newspapers in Korea on 12 dates (see the gray bars, including that for 18 Feb, in Fig. 1).

### Data analysis

In this study, a content analysis method was utilized to investigate graph usage in Korean newspapers covering COVID-19. Content analysis is a research method that uses a set of procedures to make valid inferences from text (Weber, 1990). We considered content analysis to be an appropriate methodology for this study, which investigates the use and misuse of graphs in news stories, because it enables the description of trends in communication content and the analysis of the communication content according to certain objectives (Berelson, 1952).

To answer the first research question, regarding the aspects of graph use, we coded news stories from seven daily newspapers on each focal date according to the topic of the story, based on whether or not a graph was included, the type of graph used, and the level of interpretations of the graph.

In the first phase, the news stories were coded according to the inclusion of graphs to determine the number of stories with graphs. Here, a graph refers to any type of graphical display in which n-dimensional information is conveyed by the position of points, lines, or areas on a two-dimensional image (Fry, 1983). A table also becomes a graph if it is organized based upon the frequency of data and is not just displayed raw data.

In the second phase, the collected news stories were coded in sections to examine the topics of the news stories that used graphs. Korean media outlets categorize their news into politics, economy, society, life, the world, IT/science, and opinion sections, and this study classified news stories based on these categories. The sports section, which includes news stories related to the postponing of the Tokyo Olympics, was also added to the categories and coded.

In the third phase, news stories were coded according to the type of graphs used. The graph classification system based on BS 7581:1992 (BSI, 1992), which presents international formal consensus standards, was used. BS 7581:1992 is a guidance document on methods of presenting information in tabular or graphic form in ways that allow the reader to extract information quickly and easily. Its purpose is to help those engaged in the design of tables and graphs for general use, whether in government, business, research, or the media, choose a suitable form of presentation and use it effectively (BSI, 1992). BS 7581:1992 classifies graphs as follows: single bar graph, stacked bar graph, line graph, area graph, pie graph, isotype graph, scatter graph, histogram, histogram plotted as bar graph, three-dimensional graph, superimposed graph, thematic map, illustrated graph, and pictorial graph. We added spider web graphs and band graphs to the classification system, although there were few news stories with these two types of graphs. The spider web graph was included in our analysis since it might have meaningful implications; it was used in news stories on the election of the Korean National Assembly members, which was safely conducted without changing the number of new confirmed cases despite the pandemic. The band graph was introduced in the Korean mathematics curriculum as an effective tool for representing ratios graphically.

In the last phase, to examine the level of reading graphs in news stories, news stories were coded according to the three levels of graph comprehension proposed by Friel et al. (2001). As they had characterized the level of graph comprehension corresponding to the graph readers’ ability to derive meaning from graphs created by others or by themselves, we diagnosed the level of reading graphs that news story producers exhibited in the written interpretations of the graphs in news stories using the framework of Friel and her colleagues (Friel et al., 2001). The first level is “reading the data,” where the reader obtains specific information by reading a specific value from the graph. The reader extracts the information from the data. The second level is “reading between the data,” where the reader identifies relationships shown in the graph. For instance, one should be able to identify the difference between two bars or sets of icons or to add several slices on a pie chart. In this level, readers compare the sizes of two or more datapoints or calculate the sums and differences of data. The third level is “reading beyond the data,” where the reader compares the slopes of two graphs or infers or predicts an outcome by reading trends based on the data. We coded the interpretation levels of the graphs in news stories through cross-coding and review among coders. Initially, the intercoder agreement was 90.2%, but after several discussions and reiterative coding, the intercoder agreement reached 100%.

To examine the second research question, we analyzed only news stories covering COVID-19 using graphs. The analysis was conducted based on the general types of bias in statistical graphs presented by Dick (2015) as follows: truncated axis, irregularity in scale, bias in icons, labels, misleading language, boundary bias, inconsistent placement of labels or symbols, and lack of labeling on axes. Additionally, cases of graph misuse that were considered to have mathematical implications but were not included in the inventory of Dick’s (2015) research were included in the analysis. Among all these cases, we focused on those in which graphs are misused or biased due to mathematical errors. Accordingly, open coding was performed according to the scaling method used in the graph. An inventory of graph misuse was derived, and irregularities in axis scaling and inaccuracies in scaling specifiers, which are two types of scaling misuse involving mathematical errors, were selected to code the news stories that contained graphs. Even though the inventory of Dick (2015) already included these two types of scaling errors, we tried to examine these misuses from a mathematical perspective and derive implications for mathematics education.

## Results

### Use of graphs in COVID-19 news stories

We quantitatively and qualitatively analyzed the types of graphs used and interpreted in the 5924 news stories from the seven major daily newspapers in Korea, published on the same and subsequent day of each of the six critical events, which were selected according to the changes in the number of newly confirmed cases and the spread of COVID-19 in Korea. The results are given below.

#### Quantitative aspects

Among the total of 2491 COVID-19 news stories published during the study period, 160 stories used graphs, which accounted for 6.42% of the total COVID-19 coverage. Among the non-COVID-19 stories, 113 (3.29%) out of the total of 3432 news stories used graphs. The rate of using graphs in COVID-19 stories was approximately twice as high as the rate in non-COVID-19 stories because the main issues of COVID-19 stories concerned the prevention of infectious diseases, such as testing, tracing, and isolating. Information on the status of prevention was primarily based on numerical data, such as the number of cases that were newly confirmed or released from quarantine.

**Fig. 2:**
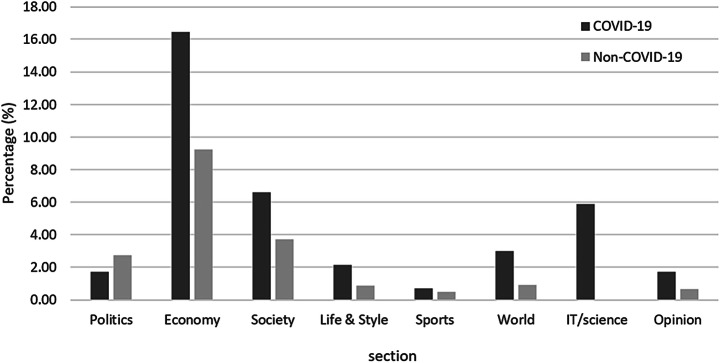
Percentage of news stories involving graphs, by section

The number of graph news stories per section was analyzed to determine which sections had mostly graph news stories. Figure 2 shows the ratio of the distribution of graph stories in the politics, economy, society, life and style, sports, world, IT/science, and opinion sections, among the COVID-19/non-COVID-19 news stories. Except for the IT/science section, both the COVID-19 and non-COVID-19 categories had a high proportion of news stories using graphs in the economy and society sections. In the economy section, graphs that indicated changes related to stock prices were mainly used in news stories. In the society section, graphs were mainly used to show population trends representing a social phenomenon or educational expenses. Particularly for COVID-19 stories in the society section, there were many news stories using graphs to represent numerical data. In the world section, the ratio of using graphs was over two times higher in COVID-19 stories than in non-COVID-19 stories. With the worldwide spread of COVID-19, graphs were mainly used in the world section to effectively communicate the status of the global outbreak of COVID-19, such as the number of confirmed cases, deaths, and testing numbers for each country. Twelve out of 13 COVID-19 graph stories covered the global outbreak of COVID-19. One interesting observation was that all graph stories in the IT/science section were COVID-19 related. We inferred that the reason for this high ratio was that the change in everyday lifestyle from face-to-face to online due to COVID-19 had dramatically affected IT businesses and the game industry, and graphs have mainly been used to communicate this change effectively. Finally, among the non-COVID-19 news stories, a relatively higher proportion of graphs was presented in the politics section. This is related to the unique circumstances of Korea. In Korea, despite the outbreak of COVID-19, the 21st National Assembly election was held in April 2020. Concerning this election, graphs showing the prior popularity rate and the election voting rate were frequently used in news stories.

Next, we analyzed the types of graphs used in 273 news stories, including 160 COVID-19 stories using graphs and 113 non-COVID-19 stories using graphs. Table 1 shows the frequency of graphs in COVID-19/non-COVID-19 news stories according to the graph classification system. The following characteristics were identified in the graphs used in COVID-19 news stories: First, bar graphs and line graphs were the two main types of graphs used in COVID-19 and non-COVID-19 news stories. Unlike non-COVID-19 stories, in which bar graphs were used more than line graphs, line graphs were used more than bar graphs in COVID-19 news stories. The elementary school mathematics curriculum of Korea introduces the bar graph as being suitable for representing discrete variables such as the number of people and the line graph as being suitable for representing the pattern of data changing over time (Ministry of Education, 2015). The line graph, which is used to represent the change in the epidemic-related data over time, was used more in COVID-19 news stories than in non-COVID-19 news stories. Second, COVID-19 news stories used pictorial graphs 8.78% more than non-COVID-19 stories. Twenty out of 25 pictorial graphs were used to report COVID-19 infection status by country and region. Pictorial graphs are effective in helping readers to intuitively comprehend information, as the graph depicts the object of comparison. Third, in the COVID-19 news stories, pie graphs were used 2.45% more than in non-COVID-19 stories. Pie graphs are a commonly used and effective type of graph for displaying proportional information (Gillan & Callahan, 2000). The mathematics education curriculum in Korea also proposes the use of pie graphs for the simple and clear representation of the ratio of each part to the whole (Ministry of Education, 2018). In the COVID-19 graph stories, pie graphs were used to express survey results concerning social phenomena changed by COVID-19. Fourth, spider web graphs and band graphs, which are not included in the BS 7581:1992 graph classification system, were used in Korean news stories. Spider web graphs were used in Korea to visually show the strengths and weaknesses of each candidate in news stories related to the general elections held in April. Four out of six COVID-19 news stories used a band graph to illustrate the composition details of confirmed cases or to represent the results of collected data for easy comparison, such as data related to the use of government subsidies to boost economic activities and data related to the number of confirmed cases. In the Korean curriculum, the band graph and pie graph are taught in sequence, and the characteristic of a band graph is described as facilitating comparisons between items. Apart from the above-described graphs, the isotype graph, representing demographic information, and the thematic map, representing a particular theme connected with a specific geographic area, are graphs related to a specific context. These types of graphs were used to represent the relationship between the number of masks and the population as well as the number of confirmed cases by region using maps in COVID-19 news stories. Accordingly, although the number of COVID-19 stories that used thematic maps and isotype graphs was small, a variety of graphs were used. We can think of two reasons for this finding. First, these graphs can represent not only the distribution of data but also the properties of data, such as the population. Second, graphs enable data to be represented more intuitively, using differences in brightness in addition to geometrical ratios, which are often restricted by the available space.

**Table 1:** Number of graphs used in news stories according to graph type

	Table	Bar graph	Line graph	Pie graph	Pictorial graph	Superimposed graph	Isotype graph	Thematic map	Spider web graph	Band graph	Total
COVID-19	15 (19)	58 (75)	53 (87)	12 (16)	14 (25)	2 (2)	1 (1)	1 (1)	0 (0)	4 (6)	160 (232)
Non-COVID-19	13 (18)	46 (63)	37 (51)	5 (7)	2 (3)	5 (5)	0 (0)	0 (0)	2 (2)	3 (8)	113 (157)
Total	28 (37)	104 (138)	90 (138)	17 (23)	16 (28)	7 (7)	1 (1)	1 (1)	2 (2)	7 (14)	273 (389)

*The number in parentheses indicates the number of graphs, considering duplicate cases in a single news story

#### Qualitative aspects with a focus on the level of reading graphs

The quantitative analysis of the types and frequency of graphs used in news stories provided an overview of the types of graphs used in COVID-19 news stories. We now focus on how graphs are used in COVID-19 news stories. The way a story makes use of graphs is related to how the news story interprets the graphs, and this relationship is derived via qualitative analysis of the content of news stories containing graphs. To this end, the three levels of graph comprehension (Friel et al., 2001) that were proposed to identify the complex nature of the public understanding of graphs were used. A total of 180 COVID-19 graph stories and 127 non-COVID-19 graph stories were analyzed according to the framework presented by Friel et al. (2001). The framework, which was used as a criterion for classifying the level of reading graphs according to readers’ responses to a given task, was adopted in this study as a criterion for classifying the level of reading graphs according to the content of news stories that included graphs. Table 2 shows the results of analyzing the level of reading graphs in COVID-19 and non-COVID-19 news stories.

**Table 2:** Number and percentage of each level of reading graphs

	Reading the data	Reading between the data	Reading beyond the data	Total
COVID-19	112 (70.00%)	36 (22.50%)	12 (7.50%)	160 (100%)
Non-COVID-19	68 (60.18%)	37 (32.74%)	8 (7.08%)	113 (100%)

In both COVID-19 and non-COVID-19 news stories, the ratio of cases that interpreted graphs at the level of “reading beyond the data” was the lowest. This means that news stories do not infer or predict trends for various phenomena through extrapolation based on graphs. Most of the graph stories are described at the level of “reading the data,” such as determining the number of confirmed COVID-19 cases through point reading, which refers to the extraction of elementary information from a graph. Moreover, at the level of “reading between the data,” readers perceived changes in the number of confirmed COVID-19 cases by reading the difference between two or more points in a single graph. Given that the more factors or variables are involved in a phenomenon, the more difficult it is to predict, it is believed that the challenge in interpreting a graph at the level of “reading beyond the data” is related to the sudden development of the COVID-19 outbreak. We found 12 news stories that interpreted graphs to predict changes that COVID-19 would bring to society in multiple ways, which meant utilizing graphs at the level of reading beyond the graph. These news stories include a story comparing the trends in confirmed cases by country, a news story analyzing trends in the price of masks with regard to the development of the COVID-19 situation, and nine stories presenting economic outlooks based on graphs concerning economic conditions. For example, there was a news story comparing the trends of confirmed COVID-19 cases between the USA and Italy based on graphs (see J. B. Hwang, 2020). Another news story used graphs to describe the change in the Korea composite stock price index and interest rates when a pandemic occurred in the past, comparing the slopes and presenting a prediction of a significant fluctuation in the index due to the COVID-19 outbreak (Jang, 2020).

In all the news stories containing graphs, the ratio of news stories interpreting graphs at the level of “reading the data” was the highest, and it was 9.82% higher in COVID-19 stories than in non-COVID-19 stories. This higher ratio is attributed to the rapid spread of COVID-19, which led Korean COVID-19 news stories to focus mainly on the prompt and accurate communication of data such as the numbers of confirmed cases, recovered cases, and deaths as a result of COVID-19 (see Hong, 2020). At the same time, this implies that the news stories provided the public with only a low level of analysis and did not help them understand the relationships in the data, the comparative analysis of graphs, or predictions based on graphs. When interpretation, such as determining the trends of current phenomena, is left to reader speculation in an urgent situation such as that of COVID-19, the public may draw various individual interpretations, such as a slowdown or an increase, and these varying interpretations may lead to public preventive measures that are not effectively united. As mentioned in the “Introduction” section, when awareness surrounding COVID-19 is not shared by the members of a community, it is difficult to put united preventive actions into practice at the community level, and this may lead to the perpetuation of the pandemic. Therefore, COVID-19 stories need to provide trend analysis and prediction through graphs, such as by showing a flattening curve to describe the controllability of the epidemic with regard to the national healthcare system capacity. In another example, a news story in the Guardian (Oltermann, 2020) presented the predicted risk of secondary spread using a graph showing the basic reproduction numbers of COVID-19. This type of professional graph analysis implies that it is not only the responsibility of the person who writes the news story to provide suitable graph news stories; the opinions of expert groups also need to be gathered. At the very least, COVID-19 news stories that can have a direct impact on the public’s decision-making behaviors concerning disease prevention should employ the level of “reading beyond the data” and provide trend analysis and prediction using graphs.

### Misuse of graphs in the coverage of COVID-19

Journalistic freedom is founded on the freedom of judgment, not on remaining neutral among conflicting values (Rawls, 1993). Therefore, news stories covering COVID-19 can be oriented toward the interpretation of a certain phenomenon and can provide value through graphs. We reviewed graph stories covering COVID-19 and identified three types of cases where news stories misused graphs. One of the misuse types involved using a line graph to represent discrete variables. From the mathematics education perspective, a line graph should be used only for continuous variables. However, from a journalistic point of view, the risk of misinterpretation of the graph by readers was low, even though it was not the right mathematical graph for the respective type of representing data. Thus, we labeled this case as negligible misuse. We discuss below the other two type of cases, *irregularity in axis scaling* and *inaccuracy in scaling specifiers*, in which graphs might cause bias in readers by presenting information in a mathematically erroneous manner.

#### Irregularity in axis scaling

**Fig. 3:**
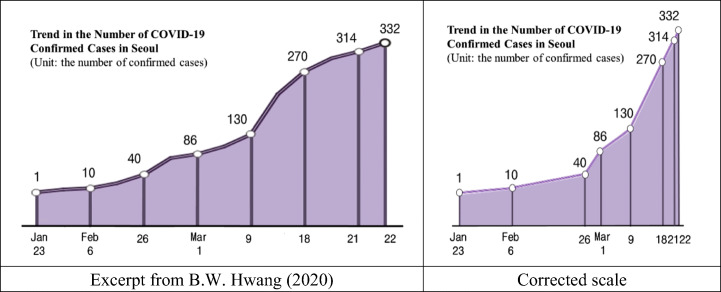
Inaccurate scaling on dates and the corrected scale

In the representation of graphs, the change of scale has a decisive effect on the slope of the graph (Zaslavsky, Sela, & Leron, 2002). A graph of the same data can have a gentle slope or a steep slope depending on the scale setting, which can deliver opposite messages to the reader. In news stories, graphs can be used according to a specific scale, which depends on the direction of interpretation. However, using an inconsistent scale is mathematically an error. Irregularity of scale was mainly found in line graphs, given that line graphs connect the values of the dependent variables that correspond to each value of the independent variables with lines so that the display can be visualized in dramatically different ways depending on the scale. Fourteen cases (16.1%) out of the 87 line graphs used in COVID-19 stories showed irregularities in scale. The left side of Fig. 3 is a graph of the number of daily cumulative confirmed cases in Seoul used in a news story on the trend of confirmed cases of COVID-19 in Seoul. We will examine how irregularity in the scale can induce bias in Fig. 3.

The left side of Fig. 3 shows the number of cumulative confirmed cases on a specific date using a line graph. Even though the dates labeled on the graph’s *x*-axis are arranged at equal intervals, except for the last interval, the actual temporal intervals between the dates are not constant. The first interval is 14 days, the second interval is 20 days, and the last interval is only 1 day. These irregular intervals constitute a misuse of axis scaling in that the interval between the selected dates is not equal. When the irregular scaling of the *x*-axis is corrected, the graph is transformed as shown on the right side of Fig. 3. In the graph on the left, the slope of the graph increases continuously over time, and the slope becomes gentle as it reaches the final point. The graph on the right shows a gradual increase in slope, and then the slope of the graph increases rapidly after March 9th. The actual rate of change of the number of confirmed cases for each date interval is approximately 15.5 cases/day from March 9th to March 18th, approximately 14.6 cases/day from March 18th to March 21st, and 18 cases/day from March 21st to March 22nd, showing a rapid increase in the last interval. In other words, around March 22nd, when the news story was written, even though the number of confirmed cases was increasing rapidly, the graph on the left of Fig. 3 made it appear as if the slope of the number of confirmed cases was decreasing. Along with the graph, the news story reported that “3 out of 10 confirmed cases on March 22nd comprised incoming travelers from France, Germany, etc. Among those confirmed, there is a steep rise in the confirmed cases from overseas travelers.” This contrasts with the message that the readers would be led to infer from the slope of the graphs. In this case, readers may interpret the graph and news story together and infer that although the rate of change of the number of confirmed COVID-19 cases is decreasing in Seoul, the number of confirmed cases from overseas is hindering this decreasing trend. In summary, this graph mitigates the actual trend of the phenomena obtained from the raw data through graph misuse, namely, irregularity of scale.

#### Inaccuracy in scaling specifiers

Specifiers, which are structural components shared via graphs, are visual dimensions that are used to represent data values (Friel et al., 2001). When drawing graphs, graph designers must consider the consistency of the ratio between the size of the data to be represented and the size of the specifiers displayed on the graph. For example, when drawing a bar graph, if the size of the data value doubles, the length of the bar must also double. In analyzing a graph, we can approach the differences in the data through the size relationships among the given specifiers. Inaccurate scaling causes inconsistencies between data differences and specifier differences. In the case of the COVID-19 graph news stories reviewed, 22 (29.3%) out of 75 bar graphs and 11 (44%) out of 25 pictorial graphs showed an inconsistency in the ratio between data values and the ratio between specifiers.

**Fig. 4:**
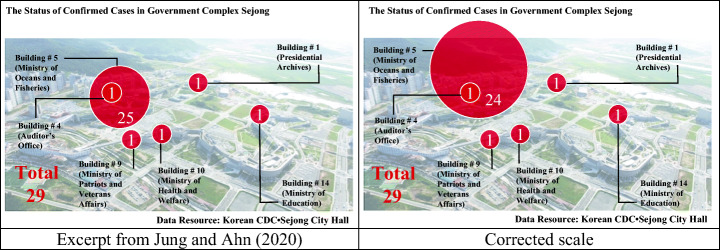
Inaccuracy in scaling specifiers and the corrected scale

The left side of Fig. 4 is a pictorial graph from a news story dated March 14, 2020, concerning the status of confirmed COVID-19 cases relating to central government officials working at the Government Complex-Sejong. At the time, in the Government Complex-Sejong, the number of confirmed cases rose rapidly to 29 within 6 days of the first confirmed case being reported in the Ministry of Health and Welfare. In particular, officials in the Ministry of Oceans and Fisheries reported 25 confirmed cases, implying a heavy concentration, considering that 29 cases in total were reported within the government complex. If the number of confirmed cases in each building is represented by a circle, the dimensions of the circles are the specifier, so the numbers of confirmed cases should have the same ratios as the dimensions. In this news story, since the Ministry of Oceans and Fisheries reported 25 cases and the rest of the departments reported one each, the ratio of the area of the large circle and the small circle should have been 25:1, which means that the ratio of the radii should have been 5:1. The corrected pictorial graph, according to this scaling of the specifiers, is shown on the right side of Fig. 4. If the graph is drawn according to the actual scale, the size of the circle representing the number of confirmed cases in the Ministry of Oceans and Fisheries appears more significant. Hence, the concentration of the confirmed cases in the Ministry of Oceans and Fisheries is emphasized more in the graph. In other words, this news story minimizes the concentration of confirmed cases in the Ministry of Oceans and Fisheries by misusing the scaling of the specifier. If the scaling of the specifier is changed arbitrarily, as shown in this case, some phenomena may be presented in a more exaggerated manner or a more diminished manner. When drawing any graph, it is necessary to clearly understand the main scale, namely, the length, width, or angle, of the specifier that expresses the data and represents the information according to the data ratio.

Another interesting point in the pictorial graph presented in Fig. 4 is that some representative circles overlap. A circle within another circle brings to mind the concept of a subset in set theory. According to the context of the news story, 24 of the 25 confirmed cases who worked for the Ministry of Oceans and Fisheries worked in one building, and 1 of the 25 confirmed cases worked in another building. Therefore, it seems that the designer of the graph attempted to represent this information with a pie graph. However, in this pictorial graph, if the specifier represents the unit of a building of affiliation, the large circle must be expressed as 24 to be correct. Otherwise, if the specifier represents the unit of a department, a small circle cannot be included in a large circle. As seen from this case, an inconsistency in the specifier setting may lead to unintended questions and misunderstandings on the part of the readers.

## Concluding remarks

This study identified the characteristics of graph use and misuse in news stories presented in Korean newspapers dealing with COVID-19 pandemic issues using quantitative and qualitative analyses. The role of news media in delivering information to the public via graphs is important when unusual situations such as the COVID-19 outbreak take place. Accordingly, we discuss the implications of these results for the future direction of graph education in school mathematics.

The majority of the graphs covered in news stories are related to the domain of statistics education. Our study revealed that graphs used in real-world news stories are often outside the scope of the mathematics taught in schools. The tables, bar graphs, line graphs, pie graphs, pictorial graphs, and band graphs that were used in Korean newspaper stories are included as part of the Korean mathematics curriculum. However, isotype graphs, thematic maps, and spider web graphs are not covered in the Korean mathematics curriculum. The curriculum of school mathematics contains some of the graph classification systems used in infographics, BS 7581:1992. The graphs taught in school mathematics only deal with types of single graphs that can represent the ratios in given data via standard geometric quantities, such as length, width, and size. The graphs used by practitioners (such as newspapers) are different in that various nongeometric differences such as differences in brightness of color are used or a variety of compound graphs are used that group heterogeneous data together. We believe that these findings should lead to a conclusion concerning not the expansion of the scope of the graphs covered in school mathematics but changes in the way graphs are approached in school-level mathematics. In essence, graphs are also a type of data representation, and these representations can be processed and transformed as much as needed for efficient delivery and interpretation of data, depending on the context. Therefore, in school mathematics, graphs should be approached as one of many tools that can represent data rather than as a fixed frame representing data. Furthermore, using an approach of comprehending graphs in context is more effective than teaching all the types of graphs that can be used. Given that all statistics are within a context (Langrall, Makar, Nilsson, & Shaughnessy, 2017), the focus of statistical graph education in school mathematics needs to be on the contextual understanding of graphs.

Consideration of understanding graphs in context is also found in previous studies. The level of “reading beyond the data,” the highest level among the three levels of reading graphs presented by Curcio (1989), or the ability to be aware of one’s relationship to the context of the graph with the goal of using interpretation to make sense of what is presented by the data in the graph and to avoid personalization of the data, which is the highest level of ability among behaviors associated with graph sense proposed by Friel et al. (2001), emphasize the inclusion of the context that the graphs are used in as well as the graphs themselves. We use these same ideas. On the other hand, the COVID-19 news stories showed the lowest rate of interpreting graphs at the highest level, “reading beyond the data,” and considering that the media producers also experienced school mathematics, there is an increasing need to include the context, which is central to “beyond the graph” reasoning, as a focus of graph education in school-level mathematics.

We have stressed that the contextual understanding of graphs is in line with the critical understanding of graphs. As in the cases reviewed in the section on the misuse of graphs in news story coverage of the COVID-19 issue, there are some suspicious news stories that induce an interpretation in a certain direction when we read the graph including the context, while these would be considered to be simple mathematical errors or negligence errors if we had read only the graph itself. Subjectivity in news content is inevitable in that everything is perceived through the subject’s experiential lens, and the news of the media outlet we encounter is also the result of the perspective of the news producer. According to Dick (2015), it is also true that the purpose of infographics in UK daily newspapers was to perpetuate their key decision-makers’ unique view of how the world should be. Therefore, media users need to know that the interpretation provided by the news embodies their intentions and that these intentions are reflected by media producers through various types of devices, even by graphs, which are often believed to provide the most objective figures. For instance, if we only focused on the graphs themselves, we would not be able to find anything more than the irregularity in scale in the news stories shown in Fig. 3. Only when the graph is approached with the context of the news story in which it is used can we discover the hidden intention to give the impression that the increase in the number of confirmed cases is slowing down by making the slope of the later part of the graph gentler with scale manipulation. This knowledge can be obtained not by looking into graphs alone but by understanding the context in which graphs are used, which means critical appraisal of the given information. Additionally, when the rational judgment of each member of the community is directly related to the safety of the community, such as in the case of COVID-19, having a critical understanding of the given information becomes even more important. In school mathematics, by emphasizing the understanding of graphs, including contexts, students can develop a critical understanding of graphs and cultivate the competencies of critical citizenship associated with the foundation of many of the decisions made in society today (Wallman, 1993; Watson & Callingham, 2003).

This study also suggests that not only do media consumers need to be better prepared to deal with graphs but also journalists have to be better trained to interpret those graphs in a manner that results in higher levels of comprehension by their audiences. Such preparation and training could be started from school-level mathematics.

In a broad sense, this study aligns with previous research on the significance of an individual’s graph literacy to understand graphically presented information, which is essential in everyday life (e.g., Galesic & Garcia-Retamero, 2011; Okan, Galesic, & Garcia-Retamero, 2016; Okan, Garcia-Retamero, Cokely, & Maldonado, 2012). However, our argument departs from other research not only in that it discusses a real-life context such as the pandemic phenomenon (unlike the artificial contexts of school mathematics) but also in that it points out the discrepancy between the graph literacy required for media usage and the graph literacy required in mathematics education. The rigid graphs dealt with in school mathematics are not encountered in the real world because they are in artificial and refined situations. Technological aids would be one way to connect the graphs used in school mathematics with graphs found in the real world by giving young students better opportunities to work with raw data of the real world that is beyond their handling capacity. In this way, future research could systematically investigate the connection between these discrepancies to broaden the field of mathematics education.

## References

[CR1] Ancker JS, Senathirajah Y, Kukafka R, Starren JB (2006). Design features of graphs in health risk communication: A systematic review. Journal of the American Medical Informatics Association.

[CR2] Berelson, B. (1952). *Content analysis in communication research*. New York, NY: Free Press.

[CR3] Bertin, J. (1983). *Semiology of graphics* (2nd ed., W. J. Berg, Trans). Madison, WI: University of Wisconsin Press.

[CR4] BSI (British Standards Institute). (1992). *BS 7581:1992 guide to presentation of tables and graphs*. London, UK: author

[CR5] Carswell CM, Burns B (1992). 16 Reading graphs: Interactions of processing requirements and stimulus structure. *Advances in Psychology: Vol. 93. Percepts, Concepts and Categories: The representation and processing of information*.

[CR6] Chen, X., & Qiu, Z. (2020). *Scenario analysis of non-pharmaceutical interventions on global COVID-19 transmissions*. arXiv preprint arXiv:2004.04529.

[CR7] Curcio FR (1981). The effect of prior knowledge, reading and mathematics achievement, and sex on comprehending mathematical relationships expressed in graphs (Doctoral dissertation, New York University, 1981). Dissertation Abstracts International.

[CR8] Curcio, F. R. (1981b). *The effect of prior knowledge, reading and mathematics achievement, and sex on comprehending mathematical relationships expressed in graphs* (Final Report). Brooklyn, NY: St. Francis College. (ERIC Document Reproduction Service No. ED 210 185)

[CR9] Curcio FR (1987). Comprehension of mathematical relationships expressed in graphs. Journal for Research in Mathematics Education.

[CR10] Curcio FR (1989). *Developing graph comprehension: Elementary and middle school activities*.

[CR11] Dick M (2015). Just fancy that: An analysis of infographic propaganda in The Daily Express, 1956-1959. Journalism Studies.

[CR12] Friel SN, Curcio FR, Bright GW (2001). Making sense of graphs: Critical factors influencing comprehension and instructional implications. Journal for Research in Mathematics Education.

[CR13] Fry, E. (1983). *A theory of graphs for reading comprehension and writing communication*. New Brunswick, NJ: Rutgers University. (ERIC Document Reproduction Service No. ED 240 528)

[CR14] Galesic M, Garcia-Retamero R (2011). Graph literacy: A cross-cultural comparison. Medical Decision Making.

[CR15] Gillan DJ, Callahan AB (2000). A componential model of human interaction with graphs: VI. Cognitive engineering of pie graphs. Human factors.

[CR16] Gracia-Retamero R, Cokely ET (2013). Communicating health risks with visual aids. Current Directions in Psychological Science.

[CR17] Gracia-Retamero R, Cokely ET (2017). Designing visual aids that promote risk literacy: A systematic review of health research and evidence-based design heuristics. Human Factors.

[CR18] Hong, S. H. (2020). Disinfecting dense sites – Local government and call center, for instance. *The Dong-ailbo*, Retrieved from https://news.naver.com/main/read.nhn?mode=LSD&mid=sec&sid1=102&oid=020&aid=0003274485.

[CR19] Hopkins, J. (n.d.). *Coronavirus resource center*. Retrieved May 20, 2020 from https://coronavirus.jhu.edu/map.html/.

[CR20] Hwang, B. W. (2020). More than 50 confirmed cases of coronavirus among ‘foreign inflows’ in Seoul... the beginning of a new hits?. *Seoulilbo*, Retrieved from https://news.naver.com/main/read.nhn?mode=LPOD&mid=sec&oid=081&aid=0003075932.

[CR21] Hwang, J. B. (2020). U.S. fails to quit COVID-19... ranks 2nd in COVID cases worldwide. *Hankyoreh Media*, Retrieved from https://news.naver.com/main/read.nhn?mode=LPOD&mid=sec&oid=028&aid=0002491077.

[CR22] Jang, W. S. (2020). “Pandemic has increased money anxiety” the Bank of Korea hints at the possibility of a rate cut. *Korea JoongAng Daily*, Retrieved from https://news.naver.com/main/read.nhn?mode=LPOD&mid=sec&oid=025&aid=0002983587.

[CR23] Jung, S. W., & Ahn, J. H. (2020). Preventing infection from the Ministry of Oceans and Fisheries… Sejong City Government Office order to prohibit movement between ministries. *The Chosunilbo*, Retrieved from https://news.naver.com/main/read.nhn?mode=LPOD&mid=sec&oid=023&aid=0003514817.

[CR24] Kim, S., Seo, Y. B., & Jung, E. (2020). Prediction of COVID-19 transmission dynamics using a mathematical model considering behavior changes. *Epidemiology and Health*, *42*. 10.4178/epih.e2020026 10.4178/epih.e2020026 PMC7285444 32375455

[CR25] Korea Audit Bureau of Circulations (2020). *The number of daily newspapers issued in 2019*.

[CR26] Langrall CW, Makar K, Nilsson P, Shaughnessy JM, Cai J (2017). Teaching and learning probability and statistics: An integrated perspective. *Compendium for Research in Mathematics Education*.

[CR27] McKnight CC, Kulm G (1990). Critical evaluation of quantitative arguments. *Assessing higher order thinking in mathematics*.

[CR28] Ministry of Education (2015). *Mathematics curriculum*.

[CR29] Ministry of Education (2018). *Mathematics 6-1 teachers’ guidebook*.

[CR30] Monteiro C, Ainley J (2007). Investigating the interpretation of media graphs among student teachers. International Electronic Journal of Mathematics Education.

[CR31] Okan Y, Galesic M, Garcia-Retamero R (2016). How people with low and high graph literacy process health graphs: Evidence from eye-tracking. Journal of Behavioral Decision Making.

[CR32] Okan Y, Garcia-Retamero R, Cokely ET, Maldonado A (2012). Individual differences in graph literacy: Overcoming denominator neglect in risk comprehension. Journal of Behavioral Decision Making.

[CR33] Oltermann, P. (2020). Fears grow in Germany of second wave of coronavirus infections. *The Guardian*, Retrieved from https://www.theguardian.com/world/2020/may/10/fears-rise-in-germany-over-second-wave-of-coronavirus-infections.

[CR34] Park SY, Kim YM, Yi S, Lee S, Na BJ, Kim CB, Kim JI, Kim HS, Kim YB, Park Y, Huh IS, Kim HK, Yoon HJ, Jang H, Kim K, Chang Y, Kim I, Lee H, Gwack J, Kim SS, Kim M, Kweon S, Choe YJ, Park O, Park YJ, Jeong EK (2020). Coronavirus disease outbreak in call center, South Korea. Emerging Infectious Diseases.

[CR35] Rawls, J. (1993). *Political liberalism*. New York, NY: Columbia University Press.

[CR36] Shaughnessy, J. M. (2007). Research on statistics learning and reasoning. In F. K. Lester Jr. (Ed.), *Second handbook of research on mathematics teaching and learning* (Vol. 2, pp. 957-1009). Charlotte, NC: Information Age; Reston, VA: National Council of Teachers of Mathematics.

[CR37] Spiegelhalter D, Pearson M, Short I (2011). Visualizing uncertainty about the future. Science.

[CR38] The Government of the Republic of Korea (2020). *Flattening the curve on COVID-19: How Korea responded to a pandemic using ICT*.

[CR39] Wainer H (1992). Understanding graphs and tables. Educational Researcher.

[CR40] Wallman KK (1993). Enhancing statistical literacy: Enriching our society. Journal of the American Statistical Association.

[CR41] Watson J, Callingham R (2003). Statistical literacy: A complex hierarchical construct. Statistics Education Research Journal.

[CR42] Weber RP (1990). *Basic content analysis*.

[CR43] World Health Organization (2020). *Coronavirus disease (COVID-2019) situation reports-49*.

[CR44] Zaslavsky O, Sela H, Leron U (2002). Being sloppy about slope: The effect of changing the scale. Educational Studies in Mathematics.

